# Prenatal and Perinatal Factors Associated with Infant Acute Lymphoblastic Leukaemia: A Scoping Review

**DOI:** 10.3390/cancers17030370

**Published:** 2025-01-23

**Authors:** Arantza Sanvisens, Clara Bueno, Oriol Calvete, Francesc Solé, Rafael Marcos-Gragera, Marta Solans

**Affiliations:** 1 Epidemiology Unit and Girona Cancer Registry, Catalan Institute of Oncology, Catalan Cancer Plan, Institut d’Investigació Biomèdica de Girona Dr. Josep Trueta (IDIBGI-CERCA), 17004 Girona, Spain; 2 Descriptive Epidemiology, Genetics, and Cancer Prevention Group, Josep Carreras Leukaemia Research Institute, 17004 Girona, Spain; 3 Stem Cell Biology, Developmental Leukemia and Immunotherapy Group, Josep Carreras Leukaemia Research Institute, 08916 Barcelona, Spain; 4 RICORS-TERAV Network, Instituto de Salud Carlos III (ISCIII), 28029 Madrid, Spain; 5 Centro de Investigación Biomédica en Red de Cáncer (CIBERONC), Instituto de Salud Carlos III (ISCIII), 08028 Barcelona, Spain; 6 Myelodysplastic Syndrome Group, Josep Carreras Leukaemia Research Institute, ICO-Hospital Germans Trias i Pujol, Universitat Autònoma de Barcelona, 08193 Barcelona, Spainfsole@carrerasresearch.org (F.S.); 7 CIBER of Epidemiology and Public Health (CIBERESP), 28029 Madrid, Spain; 8 Research Group on Statistics, Econometrics and Health (GRECS), University of Girona, 17004 Girona, Spain

**Keywords:** acute lymphoblastic leukaemia, infant, prenatal exposure, parental exposure, risk factor

## Abstract

Objective: Acute lymphoblastic leukaemia (ALL) is the most frequent childhood cancer. Infant ALL (<1 year) is rare, but it captures a lot of interest due to its poor prognosis, especially in patients harbouring *KMT2A* rearrangements, which have been demonstrated to arise prenatally. However, epidemiological studies aimed at identifying specific risk factors in such cases are scarce, mainly due to sample-size limitations. We conducted a scoping review to elucidate the prenatal or perinatal factors associated with infant ALL. Methods: Original articles, letters, or conference abstracts published up to June 2022 were identified using the PubMed, Web of Science, and Embase databases, and 33 observational studies were selected. Results: The study reveals several well-established associations across the literature, such as maternal exposure to pesticides and high birth weight, and outlines suggestive associations, such as parental heavy smoking, parental use of several medications (e.g., dipyrone), and maternal exposure to air pollution during pregnancy. Conclusions: This scoping review summarizes the few observational studies that have analysed the prenatal and perinatal risk factors for ALL in infants diagnosed before the age of 1 year. The results of this review highlight the lack of research into this specific age group, which merits further research.

## Simple Summary

This scoping review provides an overview of the few observational studies that have investigated potential risk factors for acute lymphoblastic leukaemia in infants. It summarises some well-established associations in the literature, such as maternal exposure to pesticides and high birth weight, and outlines suggestive associations, such as heavy parental smoking, parental use of multiple medications, and maternal exposure to air pollution during pregnancy, that merit further research. The results of this review highlight the lack of research on infant acute lymphoblastic leukaemia, which contrasts with the large number of epidemiological studies investigating risk factors in childhood.

## 1. Introduction

Acute lymphoblastic leukaemia (ALL) is the most common childhood cancer [1]. There are different subtypes of ALL, based on the specific differentiation stage where B or T-cell precursors are stalled, and on the cytogenetic/molecular diagnosis. Overall, lymphoblastic leukaemia accounts for 72% of all leukaemias, with an incidence rate of 31.1 per million person-years (34.5 in boys and 27.6 in girls), and with the highest incidence in the age group 1–4 years (64.7) [2].

Cases in infants aged <1 year are rare, with an incidence of 18.8 per million person years [2], but capture a lot of interest due to the different clinical and biological features in comparison with children aged from 1 to 14 years [3]. First, over 90% of cases are B-ALL, harbouring translocations of the histone-lysine N-methyltransferase 2A (*KMT2A*) gene formerly known as mixed lineage leukaemia gene (*MLL*) rearrangements [4]. Second, while childhood ALL shows a 5-year overall survival rate excessing 90%, infant cases associated with *KMT2A* rearrangements (*KMT2Ar*) have a very poor prognosis, with 5-year survival <30% [5]. In addition, it is hypothesized that most childhood leukaemias are caused first by an in utero genetic alteration, which is followed by further postnatal alterations [6]. However, studies in monozygotic twins and archived blood spots, together with the short latency between birth and diagnosis, indicate that a single prenatal hit may be sufficient to cause infant ALL [7]. Finally, while immune-related factors have been linked to childhood leukaemia [7,8], very little is known about the etiological drivers in infant ALL. Altogether, these observations suggest that infant ALL may represent a distinct entity with specific etiological factors.

A wide range of epidemiological studies have explored environmental exposures associated with the subsequent risk of ALL in childhood. To date, the most well-stablished risk factors include low doses of ionizing radiation in early childhood and maternal exposures to general pesticide, as recently pointed out in an umbrella review [9]. However, most of the studies have presented results according to the type and window of exposure (prenatal, perinatal, or during childhood), but few have thoroughly assessed the etiologic heterogeneity of childhood ALL by specific subtypes [10]. Particularly in infant ALL, studies aimed at identifying unique risk factor profiles are scarce, largely due to the sample size limitations. Herein, we conducted a scoping review to elucidate the association between prenatal or perinatal factors and infant ALL.

## 2. Methods

Original articles, letters, or conference abstracts published up to June 2022 were identified using the PubMed, Web of Science, and Embase databases. The search strategy is detailed in the Supplementary Materials, Table S1. In addition to the database searches, we manually reviewed the references of selected articles to identify relevant studies that might have been missed.

After duplicate removal, all retrieved articles underwent an initial level of title and abstract screening, followed by full-text screening of eligible abstracts. Only articles written in English or Spanish were considered, and authors were not contacted for additional information. The inclusion criteria were: (i) cohort, case-control, and cross-sectional studies; (ii) studies that reported the associations between prenatal exposures/perinatal features and subsequent risk of infant ALL (<1 year); and (iii) studies reporting relative risk estimation [e.g., hazard ratio (HR), risk ratio (RR), or odds ratio (OR)] with the corresponding measure of variability [95% confidence intervals (CI) or *p* value]. The following data were extracted from the selected studies: name of the first author, year of publication, country, study name and design, sample size, exposure assessment, main study findings, and covariates for adjustment.

This review was conducted following the recommendations of the Preferred Reporting Items for Systematic reviews and Meta-Analyses extension for Scoping Reviews (PRISMA-ScR) [11] The protocol has not been registered.

## 3. Results

**Figure 1:**
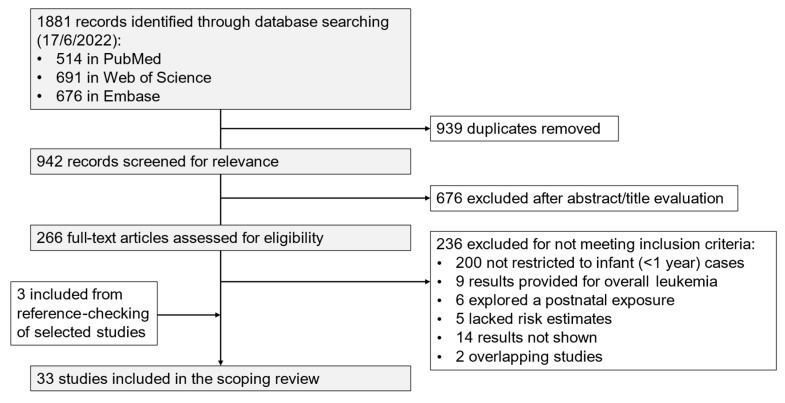
Flowchart of the study selection process.

The initial number of identified studies was 1881; finally, 33 observational studies (32 case-control studies and 1 cohort study) were selected, after duplicates had been removed and publications excluded that did not meet the inclusion criteria (Figure 1). In addition, among the studies that did not present specific results for infant ALL cases (<1 year), four analysed infant cases with less restrictive definitions (up to 24, 21, or 18 months), and 17 provided stratified results for cases diagnosed <24 or <18 months (Supplementary Material, List S1).

The main characteristics and findings of these studies are shown in Table 1. Studies from the Childhood Oncology Group (COG) [12], (n = 11), from the Childhood Leukaemia International Consortium (CLIC) [13], (n = 7), and from a multi-institutional study of infant leukaemia in Brazil [14], (n = 4), accounted for more than 60% of the studies. The former, the National Cancer Institute supported clinical trials group, is the largest organization worldwide devoted to childhood cancer research, and has conducted numerous studies, including treating ALL cases in its institutions throughout the USA and Canada. In turn, CLIC is a multinational collaboration that pools data from case-control studies on childhood leukaemia from across Europe, North America, and Australia. The latter is a collaborative study on acute infant leukaemia, supported by a network of medical centres located in 10 different Brazilian states. The rest of the studies included in this scoping review were mostly conducted in the USA or the Nordic countries.

Most of the studies did not specify the cell lineage of ALL (n = 24). However, eight studies presented specific results according to the B-cell or T-cell lineage. In addition, 12 studies presented data on *KMT2Ar*.

On the other hand, the class of examined exposures were very heterogeneous, mostly focused on maternal exposures during pregnancy but also including parental or birth characteristics. The following sections summarize the study findings according to the type of exposure.

### 3.1. Dietary Factors

In 1994, researchers from the COG hypothesized that exposure to DNA topoisomerase-II (DNAt2) inhibitors might be associated with de novo leukaemia among infants, based on the increased risk of secondary leukaemia in cancer patients with *KMT2Ar* treated with such inhibitors [48]. The most abundant environmental source of such compounds is diet; Ross et al. [15] preliminarily assessed such association within the COG study and the results, based on few cases/controls (n = 54/84), revealed no associations between a higher index of combined exposure to dietary DNAt2 inhibitors and infant ALL, but pointed out an inverse association with fish consumption and milk. Likewise, and using a larger subset of the COG, Spector et al. [16] reported, ten years later, that there was no association between a more complete index and infant ALL, regardless of *KMT2A* status. Surprisingly, the authors found an inverse association with the Vegetable and Fruit Index (VF+) (more like the index used in the previous study and characterized by high consumption of fruit and vegetables). More recently, a CLIC pooled analyses of eight case-control studies that explored the potential role of maternal coffee and tea consumption [17]. Despite the plausibility of an adverse effect from caffeine (i.e., it is an inhibitor of DNAt2 and of some tumour suppressor genes, such as *ATM* and *TP53*), no clear association with infant ALL was reported.

### 3.2. Pesticides and Other Toxic Chemicals

The reports concluding that pesticides could be risk factors for childhood leukaemia first appeared more than 25 years ago [49]. Since then, different studies have explored such associations, yielding inconsistent results mainly due to heterogeneous exposure assessment and multiple statistical testing, as recently pointed out in a systematic review and meta-analysis [50]. Based on 55 studies from over 30 countries, assessing >200 different exposures to pesticides, the authors concluded that pesticide exposure mainly during pregnancy increases the risk of ALL, particularly among infants. Mechanistic studies further support this association in infants, suggesting that transplacental exposure to mosquitocidals may cause *KMT2A* gene fusions [51].

In this review, four studies examining prenatal pesticide exposure and development of infant ALL were identified. Results from a Brazilian hospital-based case-control study revealed that children whose mothers were exposed to pesticides 3 months before conception were at least twice as likely to be diagnosed with ALL in the first year of life compared to those whose mothers were not exposed (OR: 2.1, 95% CI: 1.1; 3.9) [19]. Results by specific component yielded statistically significant associations for Permethrin, Imiprothrin, Esbiothrin, and solvents. Bailey et al. [20,21] explored data from the CLIC study regarding maternal home exposures or parental occupational exposures. In the former, the authors pooled individual-level data from 12 case-control studies and found a strong association between maternal home pesticide exposure before conception (OR: 1.44, 95% CI: 1.05; 1.97) or during pregnancy (OR: 1.9, 95% CI: 1.5; 2.3) and infant ALL. In the latter, pooled results from 13 case-control studies revealed an association between paternal exposure around conception and overall childhood ALL, yet this association did not remain in the subgroup of cases aged <1 year. Within the COG study, Slater et al. [18] investigated the potential role of several pesticides (i.e., insecticides, moth control, rodenticides, flea or tick control, herbicides, insect repellents, and professional pest exterminations), also yielding null results. It is worth mentioning a case report describing a single case of congenital leukaemia with *KMT2Ar* from a mother who heavily abused aerosolised Permethrin [52].

Other potential disrupting chemicals, such as from occupational paint exposure or household exposure to paint/stains/lacquers and petroleum products, have been explored [18,22]. Bailey et al. [22] found that home paint exposure shortly before conception, during pregnancy, and/or after birth appeared to increase the risk of childhood ALL, yet no consistent associations were reported for infant cases. In the study of Slater et al. [18], an association between gestational exposure to petroleum products (e.g., gasoline, kerosene, lubricating oils, and spot removers) was found for infant ALL without *KMT2Ar* (OR: 2.2; 95% CI: 1.0; 4.7), but not for cases of infant ALL with *KMT2Ar*.

### 3.3. Outdoor Air Pollution

In 2016, the International Agency of Research on Cancer monograph on air pollution classified its associations with childhood leukaemia as suggestive but inconsistent [53]. Since then, cumulative evidence has further explored such associations, which have been recently synthesized in a dose–response meta-analysis, revealing a potential association between outdoor air pollution and childhood ALL [54]. Studies providing specific results for infant cases, however, are very scarce, with only two USA population-based case-control studies identified in this scoping review. In 2013, Heck et al. [23] reported an almost statistically significant association between average exposure to carbon monoxide during pregnancy (based on the residence of the child at birth) with ALL diagnosed during the first year of life (OR per 1-IQR increase in carbon monoxide: 1.14, 95% CI: 0.99; 1.31). By contrast, Peckham-Gregory et al. [24] did not find an association between maternal residential proximity to major roadways, as a proxy for measuring exposure to traffic-related pollution, and infant ALL.

### 3.4. Smoking, Alcohol, and Other Drugs

Parental smoking has been suggested as a possible risk factor for childhood cancer, as chemicals in tobacco smoke are carcinogenic and can trigger DNA damage and cross the placenta. Interestingly, De la Chica et al. [55] reported that smoking 10 or more cigarettes per day (cpd) for at least 10 years and during pregnancy was associated with increased chromosome instability in amniocytes, highlighting the particular sensitivity of the 11q23 band containing the *KMT2A* gene. Many studies have explored such exposures and the subsequent risk of childhood ALL, showing inconsistent results for maternal smoking and positive associations with paternal smoking at multiple time windows [56]. In cases aged <1 year, different studies show evidence that only heavy smoking (maternal and/or paternal) may be associated with ALL [25,26,27,28,33]. In fact, the amount of daily smoked cigarettes shows a statistically significant association with ALL in two studies with the following results: (i) paternal smoking during conception time (2 years before birth) ≥15 cpd was associated with a risk of infant ALL (OR: 5.7, 95% CI: 1.5; 22.1) and this association was not observed in other age groups [28], and (ii) maternal daily consumption of 20 or more cpd during pregnancy (second and third trimester and breastfeeding) showed a statistically significant fivefold higher association (analysis available in infants <2 years) [28], though no association was observed when the cut-off was established at ≥10 cpd [25]. Evidence on alcohol consumption is much scarcer; to date, only two studies have evaluated the association between maternal alcohol consumption and infant ALL, reporting null results [26,29]. One of these also explored illicit drug use (i.e., marijuana, cocaine, and heroine), although no associations were observed [26].

### 3.5. Medication Use

Parental medication use, in association with the risk of cancer developing in offspring, has been widely studied [8]. However, only two studies have analysed the association between parental medication use during preconception and pregnancy and infant ALL. Within the COG study, Wen et al. [30] evaluated an extensive list of medications and, while not reporting statistically significant associations for cases aged <1 year, the results suggest noteworthy associations with parental use of mind-altering drugs or maternal use of antihistamines or allergy remedies. Couto et al. [31], in particular, assessed maternal use of analgesics during pregnancy in Brazil, and showed a positive association between the use of dipyrone during preconception, the first trimester of pregnancy and breastfeeding, and infant ALL, with the association being stronger in cases with *KMT2Ar*; the association between maternal use of dipyrone and *KMT2Ar* leukaemia cases was observed in all time windows [31]. Otherwise, inverse associations were reported between maternal use of acetaminophen, especially during the first trimester of pregnancy, and overall infant ALL.

### 3.6. Infections

There are currently two key hypotheses, first formulated in 1988, regarding infections and the development of childhood ALL: the ‘population mixing hypothesis’ of Kinlen [8], and the ‘delayed infection’ of Greaves [57]. Although the mechanisms differ, both authors suggest that ALL may be an abnormal response to one or more common infections acquired through personal contact. Since then, a large body of evidence has examined the association between infections and childhood ALL, reporting inconclusive findings for childhood infections [58], and positive associations with maternal infections [59,60]. However, this scoping review only identified one study providing results for infant cases [32]. Specifically, the study shows no statistically significant relationship between maternal Epstein–Barr virus and the risk of ALL in offspring, using two specific antigens (early antigen IgG and ZEBRA IgG).

### 3.7. Parental Characteristics and Reproductive History

Five studies have explored the association between parental or maternal age and infant ALL, yielding mostly null results [33,37,38,43,44]. Only in the study of Marcotte et al. [43] was an association found between paternal age <20 years and an increased risk of infant ALL (OR: 3.7, 95% CI: 1.6; 8.4). Four studies examined the potential role of parity features (e.g., history of foetal loss, etc.) or infertility (e.g., time to index pregnancy, latent class infertility, use of infertility-related drugs, etc.), reporting null associations with infant ALL [33,34,37,38]. Likewise, two studies have explored the role of maternal diseases during pregnancy and the subsequent risk of infant ALL, yielding null results for anaemia [36] and other comorbidities such as endocrine disease or diabetes, among others [33].

### 3.8. Birth Weight, Foetal Growth, and Other Perinatal Characteristics

High birth weight is among the most well-established risk factor for childhood ALL [61]. The biological mechanism behind this association remains unclear, although some authors suggest that insulin-like growth factors are involved [62]. Within this scoping review, we identified six case-control studies assessing such associations in infant cases. Except for two studies yielding null results [35,37], the remaining consistently reported strong associations between the largest categories of birth weight (i.e., >4000–4500 g [33,34,40] or ≥90th percentile [41]) and infant ALL. In addition, the study of Roman et al. [41], including 2090 infant ALL cases from three pooled case-control studies, also reported a dose–response relationship (OR per 1 kg increase = 1.2 (95% CI: 1.1; 1.3)). In contrast, foetal growth remains understudied in infant ALL cases, with only one study presenting data by age at diagnosis [42]; specifically, pooled data from 12 case-controls studies reveal that accelerated foetal growth is strongly associated with a risk of childhood ALL. Indeed, this association was observed in children whose birth weight was less than 4000 g, suggesting that accelerated foetal growth is associated with an increased risk of ALL in the absence of a high birth weight. However, no statistically significant associations were reported when restricting analyses to infant cases.

Among other birth features, congenital abnormalities were explored in a COG study including 443 infant ALL cases (excluding Down’s syndrome cases) [39]. Overall, the authors did not find any associations between eight features (cleft lip or palate, spina bifida or another spinal defect, large or multiple birthmarks, and other chromosomal abnormalities such as an unusually small head or microcephaly, rib abnormalities, urogenital abnormalities, or any other birth defect) and infant ALL. Two other studies from the COG assessed birth order, yielding different observations; one reported null findings [34], while the other found an inverse association with ALL, particularly in *KMT2Ar* cases [37]. Finally, in the study of Cnattingius et al. [33], a range of neonatal features and procedures were assessed, including physiologic jaundice, phototherapy, intrauterine or postpartum asphyxia, and supplementary oxygen, and results report mostly null associations for cases aged <1 year.

### 3.9. Caesarean Section

Increasing evidence suggests that birth by caesarean delivery affects both short-term and long-term outcomes for the neonate, including immune system development and differential microbiota colonization. Particularly in overall childhood leukaemia, current evidence points to an association between caesarean delivery and a risk of ALL [63]. However, the three studies assessing these associations in infant cases show inconsistent results. Two of these studies confirmed the association in other age groups [45,46]; however, only one of them concludes that there is an increased risk of ALL in children aged <1 year, without being able to conclude definitively whether this risk is due to prelabour or emergency caesarean delivery [47]. It should be noted that this study, unlike the others, clearly specifies which indications were considered when classifying the type of caesarean delivery, suggesting that the reason for caesarean delivery could be the key to this possible association.

## 4. Discussion

This scoping review provides an overview of the few observational studies examining potential risk factors for infant ALL. It summarizes a few well-established associations from across the literature, such as maternal exposure to pesticides and high birth weight, and outlines suggestive associations, such as parental heavy smoking, parental use of several medications (e.g., dipyrone), and maternal exposure to air pollution during pregnancy, all of which merit further research.

This study highlights the lack of research into infant ALL, which contrasts with the large number of epidemiological studies exploring the risk factors for childhood ALL. The main reasons for such scarcity are related to sample size constrains in almost all studies. While ALL is the most frequent cancer in children, cases diagnosed prior to 1 year of age are rare, with an estimated incidence of nearly 20 cases per million person-years in Europe [64] Therefore, obtaining a substantial subset of cases that allows stratified analyses by age group is difficult in case-control studies and almost unfeasible in cohort studies. Indeed, most of the included publications in this scoping review arose from the efforts of three projects (i.e., the COG, the CLIC, and the Brazilian multi-institutional study), which gathered data from multiple institutions to overcome such limitations.

During the literature search, we identified several studies with less restrictive definitions of infant cases (i.e., cases diagnosed within 18, 21, or 24 months) (Supplementary Material, List S1). The reason for widening this time window, in some Brazilian studies, was to cope with the frequent delay in the identification of ALL in some areas of Brazil [14]. However, within this scoping review, we decided to keep the strict definition as cases diagnosed during the first year of life, given the distinct biological and clinical behaviour [65]. Indeed, in more than 80% of cases, infant ALL is cytogenetically characterized by balanced chromosomal translocations involving the *KMT2A* gene at chromosome 11q23; in contrast, those rearrangements occur in ∼5% of overall childhood ALL. Multiple lines of evidence have shown that *KMT2A* rearrangements are acquired in pre-VDJ hematopoietic precursors in utero and, compared to other oncogenic fusions, initiate a strikingly rapid progression to leukaemia [66]. Given that this type of rearrangement also occurs in therapy-related acute myeloid leukaemias occurring as a complication after cytotoxic and/or radiation for a primary malignancy or autoimmune disease, such as exposure to DNAt2 inhibitors, most of the etiological hypotheses related to infant ALL occurrence are based on maternal exposure to these inhibitors. *KMT2Ar* has been described as the only genetic insult sufficient for leukemogenesis in infant ALL, in contrast to most childhood leukaemias, which are caused first by an in utero genetic alteration (‘first hit’), followed by further postnatal alterations [67,68,69,70]. Germline genetic susceptibility may also play a role, with several single-nucleotide polymorphisms correlated with ALL being identified in candidate gene or genome-wide association studies [71,72,73,74]. Overall, mechanistic studies exploring the unique molecular–genetic signature of the disease onset at the genome, epigenome, and transcriptome will help to clarify it and drive future epidemiological studies exploring environmental exposures.

Several limitations of the studies included in this scoping review must be considered. First, almost all studies are case-control studies with self-reported information on exposure. The inherent recall bias of this study design, especially regarding exposures that may be perceived harmful, should be kept in mind when interpreting their results. In addition, in most studies, collected data were not sufficiently detailed for assessing differential susceptibilities by time window, length, or magnitude of exposure, which may partly explain inconsistent findings. Moreover, exposures such as drugs, alcohol, or tobacco consumption tend to be frequently underreported, particularly during pregnancy [75]. Regarding pooled case-control studies, combining data from disparate populations may be challenging, particularly when different measurement instruments for exposure or cultural differences and variations in the prevalence of the exposures under study are involved. Therefore, despite efforts to harmonize data, misclassification issues are also expected. In addition, some studies used contextual-level data as a proxy for measuring exposure to air pollution, which unavoidably leads to some measurement error and exposure misclassification. Second, the case-control design may also introduce selection bias, given that the probability of participation in or selection for the study may be different depending on case-control status and the underlying exposures of interest. Third, ALL cytogenetic data (e.g., *KMT2Ar* status) were missing in the majority of the studies, which may be relevant when assessing etiological hypothesis. Fourth, some studies, particularly those limited to data available in existing medical records, could not adjust for important confounders, and sample size was limited, thus resulting in imprecise estimates of association.

Finally, there are some limitations related to this scoping review. Despite efforts to find all published studies and to search for conference works, findings may be affected by publication bias. In fact, published studies are limited to a few countries, and areas such as non-African or Asian countries are not represented. In addition, heterogeneity in the exposures assessed and the few studies included in each category precluded a deep qualitative summary for each exposure. Nonetheless, this study constitutes the first comprehensive overview of epidemiological studies examining aetiological risk factors for infant ALL. It has allowed us to determine the coverage of the body of literature on this topic and to identify research gaps and future research opportunities, as well as to outline areas where there is room for improvement.

## 5. Conclusions

In summary, this scoping review reveals an important research gap in the study of potential environmental risk factors involved in the aetiology of infant ALL. Maternal exposure to pesticides and high birth weight are among the most well-established conditions, while the suggestive roles of parental heavy smoking, parental use of several medications (e.g., dipyrone), and maternal exposure to air pollution during pregnancy merit further research. Given that prenatal or perinatal exposure comprises a small time window, the pivotal factors responsible may be easier to identify than for cancers with a longer latency period. New epidemiological studies should ideally gather information on cytogenetic status, as well as the time window, magnitude, and length of exposure, to counter the inconsistencies which arose in previous studies. Given the low incidence of infant ALL, this seems mostly feasible in collaborative international or pooling projects.

## Figures and Tables

**Table 1:** Summary of selected studies on infants diagnosed with ALL prior to 1 year of age.

Author, Year, Country	Study Design, Setting (Study Name)	N Cases/Controls (*KMT2A*/MLL Status)	Exposure	Exposure Assessment	Findings (OR, 95% CI) (*)
Dietary factors					
Ross et al. [15], 1996, North America	Case-control; hospital-based (COG group)	54/84	Maternal diet (potential inhibitors of DNA topoisomerase II)	Telephone interviews	Combined variable of potential dietary topoisomerase II inhibitors: low: reference; medium: 1.3 (0.4; 4.2); high: 0.5 (0.2; 1.4). Intake of fish: <1 m: reference; 1–3 week: 0.3 (0.1; 0.8); ≥4 week: 0.2 (0.1; 0.6); (*p* for trend = 0.01). Intake of milk: <daily: reference; daily: 0.3 (0.1; 0.9); no statistical differences related to intake of ice cream, margarine, butter, yogurt, cheese, eggs, beans, fresh vegetables, canned vegetables, fruit, poultry, cured meats, regular meats, soy, soft drinks, regular coffee, decaffeinated coffee, black tea, green tea, cocoa, beer, wine, or spirits.
Spector et al. [16], 2005, North America	Case-control; hospital-based (COG group)	240/255 (*KMT2A*/*MLL* status determined)	Maternal diet	Telephone interview using structured computer- assisted questionnaire and medical records, and food frequency questionnaire, self-reported	DNAt2 food index in MLL+; Q1: reference; Q2: 0.5 (0.3; 1.1); Q3: 0.7 (0.4; 1.4); Q4: 0.5 (0.2; 1.1); (*p* for trend = 0.19) DNAt2 food index in MLL-: Q1: reference; Q2: 0.7 (0.3; 1.9); Q3: 0.9 (0.4; 2.5); Q4: 1.0 (0.4; 2.8); (*p* for trend = 0.83) DNAt2 food index overall: Q1: reference; Q2: 0.5 (0.3; 1.0); Q3: 0.7 (0.4; 1.3); Q4: 0.7 (0.4; 1.2); (*p* for trend = 0.29) VF+ index in MLL+: Q1: reference; Q2: 0.6 (0.3; 1.1); Q3: 0.2 (0.1; 0.5); Q4: 0.5 (0.2; 0.9); (*p* for trend = 0.01) VF+ index in MLL-; Q1: reference; Q2: 1.2 (0.4; 3.3); Q3: 1.1 (0.4; 3.0); Q4: 1.0 (0.4; 2.9); (*p* for trend = 0.96) VF+ index overall: Q1: reference; Q2: 0.7 (0.4; 1.2); Q3: 0.5 (0.3; 0.9); Q4: 0.7 (0.4; 1.2); (*p* for trend = 0.09).
Milne et al. [17], 2018, North America, Europe, and Australasia	Pooled analysis; 8 case-control studies; multiple sources (CLIC)	93/484 (*KMT2A*/*MLL* status determined)	Maternal coffee and tea consumption	Food frequency questionnaire	Coffee consumption: none: reference; any: 0.99 (0.53; 1.88); >0–2 cups/day: 1.01 (0.53; 1.91); >2 cups/day: 0.48 (0.12; 1.90); (*p* for trend = 0.49). Tea consumption: none: reference; any: 0.57 (0.30; 1.08); >0–2 cups/day: 0.48 (0.22; 1.04); >2 cups/day: 1.57 (0.32; 7.75); (*p* for trend = 0.23).
Pesticides and other toxic chemicals					
Slater et al. [18], 2011, North America	Case-control; hospital-based (COG group)	264/324 (*KMT2A*/*MLL* status determined)	Maternal exposure to nine household chemicals (insecticides, moth control, rodenticides, flea or tick control, herbicides, insect repellents, professional pest exterminations, paints/stains/lacquers, and petroleum products)	Structured, computer-assisted telephone interviews	No associations overall among ALL and any exposure to chemicals. Petroleum products (any exposure): (yes vs. no): ALL MLL-: 2.21 (1.04; 4.67); ALL MLL+: 1.30 (0.68; 2.49). Petroleum products (exposure during pregnancy): (yes vs. no): ALL MLL-: 2.39 (1.12; 5.11); ALL MLL+: 1.23 (0.62; 2.43).
Ferreira et al. [19], 2013, Brazil	Case-control study; multicentric hospital-based (multi-institutional study of infant leukaemia)	88/254	Maternal pesticide exposure	Face-to-face interview using a standardized questionnaire	By time window: (yes vs. no): any: 2.10 (1.14; 3.86); preconception (3 months before pregnancy); 2.40 (1.20; 4.81); 1st trimester: 1.86 (0.94; 3.72); 2nd trimester: 1.75 (0.87; 3.55); 3rd trimester: 1.88 (0.93; 3.79). By specific components: (yes vs. no): Prallethrin: 1.52 (0.15; 15.32); Permethrin: 2.47 (1.17; 5.25); Imiprothrin: 2.61 (1.06; 6.93); Esbiothrin: 3.03 (1.13; 8.09); Tetramethrin: 1.56 (0.65; 3.72); D-Phenothrin: 4.16 (0.85; 20.29); D-Allethrin: 1.56 (0.65; 3.72); solvents: 2.17 (1.06; 4.43).
Bailey et al. [20], 2014, North America, Europe, and Australasia	Pooled analysis; 12 case-control studies; multiple sources (CLIC)	958/2272	Parental occupational pesticide exposure	Self-reported work; jobs assessed for pesticide exposure; pesticide structured questionnaire	Maternal exposure during pregnancy: no or minimal likelihood of exposure: reference; high likelihood of pesticide exposure: 1.20 (0.50; 2.88). Paternal exposure around conception: no or minimal likelihood of exposure: reference; high likelihood of pesticide exposure: 1.18 (0.77; 1.79).
Bailey et al. [21], 2015, North America, Europe, and Australasia	Pooled analysis; 12 case-control studies; multiple sources (CLIC)	867/2149 (*KMT2A*/*MLL* status determined)	Maternal home pesticide exposure	Telephone, self-administered, or face-to-face interview; structured questionnaires	1–3 months before conception: (yes vs. no): 1.44 (1.05; 1.97); during pregnancy: (yes vs. no): 1.87 (1.48; 2.37); after birth: (yes vs. no): 1.22 (0.91; 1.62).
Bailey et al. [22], 2015, North America, Europe, and Australasia	Pooled analysis; 8 case-control studies; multiple sources (CLIC)	485/738 (*KMT2A*/*MLL* status determined)	Maternal home paint exposure	Structured questionnaire	1–3 months before conception: (yes vs. no): 1.63 (0.94; 2.85); within the year before conception: (yes vs. no): 0.52 (0.32; 0.62); during pregnancy: (yes vs. no): 1.29 (0.99; 1.68); after birth: (yes vs. no): 1.53 (0.93; 2.52).
Outdoor air pollution					
Heck et al. [23], 2013, EEUU (California)	Case-control; population-based	81/not available	Traffic-related air pollution	Local traffic exposures for each trimester of pregnancy at the address indicated in the birth certificate	Per 1 IQR increase in carbon monoxide: 1.14 (0.99; 1.31).
Peckham-Gregory et al. [24], 2019, EEUU	Case-control; population based	105/4838	Residential proximity to major roadways	Geocoded street address of the maternal residence at time of delivery and Texas roadway network (geographic data)	Distance: (continuous); 1.00 (0.97; 1.04). Proximity to major roadway: >500 m: reference; ≤500 m: 0.79 (0.51; 1.24). Roadway density: low: reference; medium: 0.80 (0.49; 1.31); high: 0.79 (0.48; 1.30).
Smoking, alcohol, and other drugs					
Mucci et al. [25], 2004, Sweden	Cohort study; population-based	Not available	Maternal smoking	Medical birth registry	Maternal smoking (HR, 95% CI): no: reference; yes: 0.56 (0.31; 1.01); 1–9 cigarettes: 0.57 (0.28; 1.15); ≥10 cigarettes: 0.55 (0.22; 1.37); (*p* for trend = 0.071).
Slater et al. [26], 2011, North America	Case-control; hospital-based (COG group)	264/324 (*KMT2A/MLL* status determined)	Maternal smoking, alcohol, and illicit drug use	Computer-assisted telephone interviews with structured questionnaire	Cigarette use: any: 0.97 (0.62; 1.53); before pregnancy: 0.99 (0.63; 1.56); during pregnancy: 0.87 (0.54; 1.40). Alcohol use: any: 0.86 (0.61; 1.22); before pregnancy: 0.85 (0.60; 1.20): during pregnancy: 0.75 (0.49; 1.17); Illicit drug use: any: 0.84 (0.47; 1.51). Reference groups included never and ever consumers not reporting use during the relevant time periods. Similar results were obtained when only never consumers were used.
Ferreira et al. [27], 2012, Brazil	Case-control study; multicentric hospital-based (multi-institutional study of infant leukaemia)	88/255	Maternal smoking	Face-to-face interview using standardized questionnaire	Maternal smoking: (yes vs. no): 0.65 (0.31; 1.38).
Milne et al. [28], 2012, Australia	Case-control study; population-based	31/66	Parental smoking	Self-administered questionnaire	Paternal smoking: none: reference; 1–14 cigarettes per day: 1.94 (0.38; 9.82); ≥15 cigarettes per day: 5.73 (1.49; 22.09). Maternal smoking: not associated with risk of ALL; results did not vary according to child’s age.
Ferreira et al. [29], 2015, Brazil	Case-control study; multicentric hospital-based (multi-institutional study on infant leukaemia)	88/255	Maternal alcohol consumption	Face-to-face interview using standardized questionnaire	Any beverages: (yes vs. no): 1.29 (0.73; 2.27); preconception (3 previous months): (yes vs. no): 1.56 (0.88; 2.79); during pregnancy: (yes vs. no): 1.49 (0.77; 2.89).
Medication use					
Wen et al. [30], 2002, North America and Australia	Case-control; hospital-based (COG group)	64/81	Parental medication use	Telephone interview using structured questionnaire; self-reported	Mothers (99% CI): (yes vs. no): vitamins: 0.9 (0.1; 7.1); iron supplements: 1.1 (0.3; 3.9); antihistamines or allergy remedies: 4.3 (0.6; 32.1); mind-altering drugs: 6.4 (0.3; 127.0). Fathers (99% CI): (yes vs. no): mind-altering drugs: 3.0 (0.7;12.3).
Couto et al. [31], 2015, Brazil	Case-control study; multicentric hospital-based (multi-institutional study of infant leukaemia)	84/269 (*KMT2A/MLL* status determined)	Maternal use of analgesic medicines	Face-to-face interview using standardized questionnaire	Any: no: reference; Acetaminophen: 0.69 (0.27; 1.74); Dipyrone: 2.23 (1.19; 4.20); both: 0.87 (0.29; 2.65). Preconception (3 previous months): no: reference; Acetaminophen: 0.65 (0.19; 2.23); Dipyrone: 2.90 (1.58; 5.35); both: 0.49 (0.12; 2.02). 1st trimester: no: reference; Acetaminophen: 0.25 (0.10; 0.95); Dipyrone: 1.99 (1.09; 3.60); both: 0.53 (0.13; 2.15). 2nd trimester: no: reference; Acetaminophen: 0.29 (0.07; 1.13); Dipyrone: 2.01 (1.11; 3.64); both: 0.65 (0.18; 2.40). 3rd trimester: no: reference; Acetaminophen: 0.50 (0.19; 1.32); Dipyrone: 1.95 (1.01; 3.77); both: 0.65 (0.17; 2.54). Breastfeeding: no: reference; Acetaminophen: 0.51 (0.14; 1.82); Dipyrone: 2.92 (1.46; 5.86); both: 0.52 (0.09; 3.14). Similar results provided also for MLL+ cases (n = 43) except: 1st trimester: no: reference; Acetaminophen: 0.45 (0.09; 2.36).
Infections					
Tedeschi et al. [32], 2009, Iceland and Finland	Nested case-control study; population-based	~25/71	Maternal EBV infection	Maternal IgG antibodies to EBV, early antigen (EA), and EBV transactivator Zebra protein, assessed during first trimester	EA IgG: (yes vs. no): 1.4 (0.6; 3.4); Zebra IgG: (yes vs. no): 1.7 (0.4; 8.1).
Parental and perinatal features					
Cnattingius et al. [33], 1995, Sweden	Nested case-control; population-based	97/485	Infant birth weight and complications during neonatal period; maternal age at delivery, parity, history of infertility, previous abortions, smoking, and complications during pregnancy and delivery	Medical birth registry	Maternal characteristics (unadjusted model): Age at delivery (years): ≥19: 1.9 (0.7; 5.4); 20–24: 0.7 (0.4; 1.3); 25–29: reference; 30–34: 1.0 (0.6; 1.7); ≥35: 0.8 (0.4; 1.8). Parity: 1: 1.1 (0.7; 1.7); 2–3: reference; ≥4: 0.6 (0.2; 1.9). Infertility: (yes vs. no): 0.0 (0.0; 8.6). Spontaneous abortions: (yes vs. no): 2.1 (0.9; 4.8). Maternal diseases during pregnancy: (yes vs. no): endocrine diseases: 1.0 (0.1; 8.6); diabetes: 1.3 (0.1; 11.2); diseases in blood-forming organs: 2.5 (0.5; 13.6); renal disease: 2.5 (0.2; 27.6); hypertensive disease: 1.4 (0.6; 3.2). Daily smoking in early pregnancy: (yes vs. no): 1.0 (0.4; 2.1). Infant characteristics (unadjusted model): Birth weight (g): <1500: 0.0 (0.0; 5.0); 1500–1999: 0.0 (0.0; 5.6); 2000–2499: 0.0 (0.0; 2.3); 2500–2999: 1.3 (0.7; 2.8); 3000–3499: reference; 3500–3999: 1.2 (0.7; 2.0); 4000–4499: 0.8 (0.3; 1.7); ≥4500: 2.8 (1.01; 7.6). Neonatal characteristics and procedures: (yes vs. no): physiologic jaundice: 0.9 (0.3; 2.5); phototherapy: 0.9 (0.2; 4.1); intrauterine asphyxia: 1.4 (0.5; 3.7); postpartum asphyxia: 0.6 (0.2; 2.2); multiple birth: 0.0 (0.0; 2.0); supplementary oxygen: 1.8 (0.6;5.1); Down’s syndrome: 15.0 (1.6; 144.2). Adjusted model: Maternal diseases during pregnancy: (yes vs. no): diabetes: 0.0 (0.0; 8.6); diseases in blood-forming organs: 0.0 (0.0; 17.4); hypertensive disease: 0.7 (0.1; 3.4). Infant: (yes vs. no): birth weight < 1500 g: 0.0 (0.0; 5.6); birth weight ≥ 4500 g: 3.2 (0.5; 19.7); multiple birth: 0.0 (0.0; 3.2); postpartum asphyxia: 0.0 (0.0; 4.1); supplementary oxygen: 1.6 (0.2; 15.2).
Ross et al. [34], 1997, North America	Case-control; hospital-based (COG group)	181/468	Birth characteristics and maternal reproductive history	Telephone interviews	Birth weight (g): ≤3000: reference; 3001–3500: 0.99 (0.55; 1.79); 3501–4000: 1.07 (0.58; 1.98); >4000: 2.51 (1.17; 5.41). Birth order: 1st-born: reference; 2nd–3rd-born: 1.09 (0.73; 1.62); ≥4th-born: 0.85 (0.38; 1.89). Previous stillbirth: (yes vs. no): 0.85 (0.23; 3.11). Previous miscarriage: (yes vs. no): 1.33 (0.82; 2.15). Previous abortion: (yes vs. no): 1.09 (0.62; 1.89). Total foetal loss: 0: reference; 1: 1.22 (0.79; 1.90); ≥2: 0.86 (0.45; 1.64). Total prior foetal loss: 0: reference; 1: 1.34 (0.85; 2.12); ≥2: 1.03 (0.52; 2.05). N° of live births: (continuous): 0.92 (0.78; 1.08).
Hjalgrim et al. [35], 2004, Denmark, Sweden, Norway, Iceland	Case-control; population-based	57/281	Birth weight	Medical birth registries	Birth weight (g): <2500: 2.51 (0.43; 14.59); 2500–2999: 0.42 (0.12; 1.52); 3000–3499: 0.73 (0.35; 1.55); 3500–3999: reference; 4000–4499: 1.26 (0.54; 2.94); ≥4500: 2.20 (0.65; 7.50). Per 1-kg increase: 1.62 (0.89; 2.96).
Peters et al. [36], 2006, North America	Case-control; hospital-based (COG group)	115/not available (*KMT2A*/*MLL* status determined)	Maternal anaemia during pregnancy	Medical records indicating haemoglobin concentration (<11 g/dL)	Overall ALL: (yes vs. no): 1.14 (0.65; 2.01); ALL MLL+: (yes vs. no): 0.98 (0.50; 1.91); ALL MLL-: (yes vs. no): 1.33 (0.55; 3.24).
Spector et al. [37], 2007, North America	Case-control; hospital-based (COG group)	149/255 (*KMT2A*/*MLL* status determined)	Birth characteristics and maternal reproductive history	Structured computer-assisted telephone interviews	Increasing birth order: ALL: 0.56 (0.32; 0.98); ALL MLL+: 0.50 (0.25; 1.01). No associations between ALL and birth weight, maternal history of foetal loss, maternal age, gestational age, and body mass index.
Puumala et al. [38], 2010, North America	Case-control; hospital-based (COG group)	264/324 (*KMT2A*/*MLL* status determined)	Parental infertility-related factors or treatment	Telephone interview with structured questionnaire Self-reported	Latent class infertility: (yes vs. no): 1.27 (0.79; 2.05). Prior foetal loss: no: reference; 1: 1.13 (0.71; 1.79); ≥2: 1.76 (0.87; 3.59). Maternal age: (per 1 year increase): 0.98 (0.94; 1.02). Use of ovarian stimulating drugs: (yes vs. no): 1.42 (0.64; 3.15). Time to index pregnancy: not trying (*): 1.32 (0.88; 1.96); <1 year: reference; ≥1 year: 1.32 (0.76; 2.30). (*) when restricted to MLL(-): 2.50 (1.36; 4.61) No differences in MLL status from any other exposures.
Johnson et al. [39], 2010, North America	Case-control; hospital-based (COG group)	264/324 (*KMT2A*/*MLL* status determined)	Congenital abnormalities	Telephone interview with structured questionnaire; self-reported	Any congenital abnormality: (yes vs. no): 1.4 (0.8; 2.3); large or multiple birthmarks: (yes vs. no): 1.5 (0.8; 3.0); urogenital systema abnormality: (yes vs. no): 0.8 (0.2; 2.6); other congenital abnormalities: (yes vs. no): 1.5 (0.7; 3.3); no differences by sex.
Bjørge et al. [40], 2013, Denmark, Finland, Norway, and Sweden	Nested case-control study; population-based	Not available	Birth weight	Obtained from birth registries	Risk of ALL was elevated in children with birth weight > 4000 g in all age groups.
Roman et al. [41], 2013, Germany, UK, and EEUU	Pooled analysis; 3 case-control studies	2090/5107	Birth weight	Face-to-face interview	According to centile: <10: 0.7 (0.6; 0.9); 10–19: 1.0 (0.8; 1.2); 20–79: reference; 80–89: 1.0 (0.8; 1.2); ≥90: 1.2 (1.0; 1.4). Per 1 kg increase: 1.2 (1.1; 1.3).
Milne et al. [42], 2013, North America, Europe, and Australasia	Pooled analysis; 12 case-control studies; multiple sources (CLIC)	636/1447 (LGA) 179/268 (POBW)	Foetal growth	Questionnaire and medical records	Large for gestational age (LGA): (yes vs. no): 1.04 (0.75; 1.44). Proportion of optimal birth weight (POBW): (yes vs. no): 1.09 (0.87; 1.37).
Marcotte et al. [43], 2017, EEUU	Case-control; population-based	219/45,392	Parental age	Birth record information	Maternal age; ≤19 years: 0.51 (0.24; 1.09); 20–24 years: 0.77 (0.47; 1.24); 25–29 years: reference; 30–34 years: 1.39 (0.90; 2.13); 35–39 years: 1.35 (0.73; 2.49); 40 + years: 1.07 (0.31; 3.76). Paternal age: ≤19 years: 3.69 (1.62; 8.41); 20–24 years: 1.47 (0.88; 2.45); 25–29 years: reference; 30–34 years: 0.98 (0.63; 1.52); 35–39 years: 0.70 (0.39; 1.27); 40+ years: 1.04 (0.52; 2.07). Similar results with imputed parental age.
Petridou et al. [44], 2018, North America, Europe, and Australasia	Pooled analysis; 11 case-control and 5 nested case-control studies; multiple sources (CLIC)	272/860	Parental age	Birth/health registry data	Paternal age: (per 5-year increment): 1.09 (0.92; 1.29); heterogeneity: I^2^:0%, *p* = 0.53. Maternal age: (per 5-year increment): 0.98 (0.81; 1.18); heterogeneity: I^2^:0%, *p* = 0.53.
Caesarean section					
Marcotte et al. [45], 2016, North America, Europe, and Australasia	Pooled analysis; 13 case-control studies; multiple sources (CLIC)	1000/4143	Caesarean delivery	Questionnaire and medical records	Caesarean delivery: (yes vs. no): 1.14 (0.79; 1.64); prelabour caesarean delivery: (yes vs. no): 2.62 (0.96; 7.19).
Wang et al. [46], 2017, EEUU (California)	Case-control; population-based	592/2216	Caesarean section	Birth records	Mode of delivery: vaginal: reference; caesarean section: 0.94 (0.78; 1.14). Subset with detail of mode of delivery: vaginal: reference; caesarean section overall: 1.21 (0.88; 1.67); emergency caesarean section: 1.24 (0.69; 2.25); elective caesarean section: 1.20 (0.84; 1.71).
Marcotte et al. [47], 2018, North America	Case-control; hospital-based (COG group)	264/324 (*KMT2A*/*MLL* status determined)	Caesarean delivery	Structured, computer-assisted telephone interviews; medical records	Caesarean delivery (CD) (interview): vaginal: reference; any indication: 1.52 (1.02; 2.25); emergency CD: 1.99 (1.10; 3.59); prelabour CD: 1.41 (0.80; 2.51). Caesarean delivery (medical records): vaginal: reference; any indication: 1.89 (1.14; 3.15); emergency CD: 1.80 (0.93; 3.51); prelabour CD: 2.04 (1.00; 4.15). Duration of labour: >6–10 h: reference; 0–3 h: 1.14 (0.53; 2.44); >3–6 h: 1.05 (0.52; 2.12); >10 h: 1.46 (0.76; 2.80).

CI: confidence interval; CLIC: Childhood Leukaemia International Consortium; COG: Children’s Oncology Group; DNAt2: DNA topoisomerase II; HR: hazard ratio; OR: odds ratio; VF: vegetable and fruit. * Findings from the maximally adjusted model. Direct statistically significant associations with infant ALL are in red. Inverse statistically significant associations with infant ALL are in green.

## Data Availability

Data sharing is not applicable to this article as no datasets were generated or analysed during the current study.

## Supplementary Materials

The following supporting information can be downloaded at: https://www.mdpi.com/article/10.3390/cancers17030370/s1. Supplementary Table S1. Search strategy in (A) PubMed and (B) Web of Science and EMBASE. Supplementary List S1. List of studies that stratify results for <24 or <18 months or that include only infant cases but with less restrictive definitions (up to 24 months, up to 21 months, or up to 18 months).
