# Supplementary material for: Prenatal and Perinatal Factors Associated with Infant Acute Lymphoblastic Leukaemia: A Scoping Review
Source: Cancers (Basel). 2025 Jan 23;17(3):370. doi: 10.3390/cancers17030370 (PMC11816379; doi:10.3390/cancers17030370)
Supplement: Supplementary file 1 [file cancers-17-00370-s001.zip › cancers-3391579-supplementary.pdf]

Supplementary Table S1. Search strategy in A) PubMed and B) Web of Science and EMBASE.

A.

	Search terms
[1] ALL	"precursor cell lymphoblastic leukemia-lymphoma"[Mesh] OR "acute lymphoblastic"[tiab] OR "acute lymphocytic"[tiab]
[2] Infants (<1 year)	"infant"[Mesh] OR "infant*"[tiab] OR "child*"[tiab] OR "neonatal"[tiab] OR "newborn"[tiab]
[3] Prenatal exposure	"prenatal exposure delayed effects"[Mesh] OR "maternal exposure"[Mesh] OR "paternal exposure"[Mesh] OR "prenatal"[tiab] OR "maternal"[tiab] OR "paternal"[tiab] OR "pregnan*" [tiab] OR "parental" [tiab] OR "in-utero" [tiab] OR "birth"[tiab] OR "fetal[tiab]"
[4] Study design	"case-control studies"[Mesh] OR "cohort studies"[Mesh] OR "cross sectional studies"[Mesh] OR "case-control"[tiab] OR "cohort"[tiab] OR "cross sectional"[tiab]

<u>Search strategy MEDLINE (PubMed):</u> [1] AND [2] AND [3] AND [4]

B.

<u>Search strategy Web of Science and EMBASE:</u> ("acute lymphoblastic" OR "acute lymphocytic") AND ("child*" OR "infant*" OR "neonatal" OR "newborn") AND ("prenatal" OR "maternal" OR "paternal" OR "pregnan*" OR "parental" OR "in-utero" OR "birth" OR "fetal") AND ("case-control" OR "cohort" OR "cross sectional")

Supplementary List S1. List of studies that stratify results for <24[1-17] or <18[18] months or that include only infant cases but with less restrictive definitions (up to 24 months[19-20], up to 21 months[21], or up to 18 months[22-23])

1. Kaye, S. A., Robison, L. L., Smithson, W. A., Gunderson, P., King, F. L., & Neglia, J. P. (1991). Maternal reproductive history and birth characteristics in childhood acute lymphoblastic leukemia. Cancer, 68(6), 1351–1355. https://doi.org/10.1002/1097-0142(19910915)68:6<1351::aid-cncr2820680627>3.0.co;2-j
2. Ma, X., Metayer, C., Does, M. B., & Buffler, P. A. (2005). Maternal pregnancy loss, birth characteristics, and childhood leukemia (United States). *Cancer causes & control*, *16*(9), 1075–1083. https://doi.org/10.1007/s10552-005-0356-9
3. McKinney, P. A., Cartwright, R. A., Saiu, J. M., Mann, J. R., Stiller, C. A., Draper, G. J., Hartley, A. L., Hopton, P. A., Birch, J. M., & Waterhouse, J. A. (1987). The inter-regional epidemiological study of childhood cancer (IRESCC): a case control study of aetiological factors in leukaemia and lymphoma. *Archives of disease in childhood*, *62*(3), 279–287. https://doi.org/10.1136/adc.62.3.279
4. Naumburg, E., Bellocco, R., Cnattingius, S., Jonzon, A., & Ekbom, A. (2002). Perinatal exposure to infection and risk of childhood leukemia. *Medical and pediatric oncology*, *38*(6), 391–397. https://doi.org/10.1002/mpo.10084
5. Milne, E., Greenop, K. R., Scott, R. J., de Klerk, N. H., Bower, C., Ashton, L. J., Heath, J. A., & Armstrong, B. K. (2013). Parental alcohol consumption and risk of childhood acute lymphoblastic leukemia and brain tumors. *Cancer causes & control*, *24*(2), 391–402. https://doi.org/10.1007/s10552-012-0125-5
6. Whitehead, T. P., Wiemels, J. L., Zhou, M., Kang, A. Y., McCoy, L. S., Wang, R., Fitch, B., Petrick, L. M., Yano, Y., Imani, P., Rappaport, S. M., Dahl, G. V., Kogan, S. C., Ma, X., & Metayer, C. (2021). Cytokine Levels at Birth in Children Who Developed Acute Lymphoblastic Leukemia. *Cancer epidemiology, biomarkers & prevention*, *30*(8), 1526–1535. https://doi.org/10.1158/1055-9965.EPI-20-1704
7. Yeazel, M. W., Buckley, J. D., Woods, W. G., Ruccione, K., & Robison, L. L. (1995). History of maternal fetal loss and increased risk of childhood acute leukemia at an early age. A report from the Children's Cancer Group. *Cancer*, *75*(7), 1718–1727. https://doi.org/10.1002/1097-0142(19950401)75:7<1718::aid-cncr2820750725>3.0.co;2-g
8. Lei, U., Wohlfahrt, J., Hjalgrim, H., Hjalgrim, L. L., Simonsen, H., & Melbye, M. (2000). Neonatal level of thyroid-stimulating hormone and acute childhood leukemia. *International journal of cancer*, *88*(3), 486–488.
9. Freedman DM, Stewart P, Kleinerman RA, Wacholder S, Hatch EE, Tarone RE, Robison LL, Linet MS. Household solvent exposures and childhood acute lymphoblastic leukemia. Am J Public Health. 2001 Apr;91(4):564-7. doi: 10.2105/ajph.91.4.564. PMID: 11291366; PMCID: PMC1446651.
10. Chow, E. J., Puumala, S. E., Mueller, B. A., Carozza, S. E., Fox, E. E., Horel, S., Johnson, K. J., McLaughlin, C. C., Reynolds, P., Von Behren, J., & Spector, L. G. (2010). Childhood cancer in relation to parental race and ethnicity: a 5-state pooled analysis. *Cancer*, *116*(12), 3045–3053. https://doi.org/10.1002/cncr.25099
11. Junqueira, M. E. R., de Oliveira, C. T., Tone, L. G., Lee, M. L. M., de Andréa, M. L. M., Bruniera, P., Epelman, S., Odone Filho, V., Bonilha, E. A., de Freitas, M., Okamura, M. N., Vico, E. R., Stevens, A. P., Rabello Neto, D. L., & Wünsch

Filho, V. (2020). Caesarean sections, prenatal and postnatal conditions and childhood acute lymphoblastic leukaemia: A case-control study in the State of São Paulo, Brazil. *Cancer epidemiology*, *69*, 101851. https://doi.org/10.1016/j.canep.2020.101851

12. Shaw, A. K., Infante-Rivard, C., & Morrison, H. I. (2004). Use of medication during pregnancy and risk of childhood leukemia (Canada). *Cancer causes & control*, *15*(9), 931–937. https://doi.org/10.1007/s10552-004-2230-6
13. Nishi, M., & Miyake, H. (1989). A case-control study of non-T cell acute lymphoblastic leukaemia of children in Hokkaido, Japan. *Journal of epidemiology and community health*, *43*(4), 352–355. https://doi.org/10.1136/jech.43.4.352
14. Scélo, G., Metayer, C., Zhang, L., Wiemels, J. L., Aldrich, M. C., Selvin, S., Month, S., Smith, M. T., & Buffler, P. A. (2009). Household exposure to paint and petroleum solvents, chromosomal translocations, and the risk of childhood leukemia. *Environmental health perspectives*, *117*(1), 133–139. https://doi.org/10.1289/ehp.11927
15. Menegaux, F., Steffen, C., Bellec, S., Baruchel, A., Lescoeur, B., Leverger, G., Nelken, B., Philippe, N., Sommelet, D., Hémon, D., & Clavel, J. (2005). Maternal coffee and alcohol consumption during pregnancy, parental smoking and risk of childhood acute leukaemia. *Cancer detection and prevention*, *29*(6), 487–493. https://doi.org/10.1016/j.cdp.2005.06.008
16. Shu, X. O., Potter, J. D., Linet, M. S., Severson, R. K., Han, D., Kersey, J. H., Neglia, J. P., Trigg, M. E., & Robison, L. L. (2002). Diagnostic X-rays and ultrasound exposure and risk of childhood acute lymphoblastic leukemia by immunophenotype. *Cancer epidemiology, biomarkers & prevention*, *11*(2), 177–185.
17. Shu, X. O., Stewart, P., Wen, W. Q., Han, D., Potter, J. D., Buckley, J. D., Heineman, E., & Robison, L. L. (1999). Parental occupational exposure to hydrocarbons and risk of acute lymphocytic leukemia in offspring. *Cancer epidemiology, biomarkers & prevention*, 8(9), 783–791.
18. MacArthur, A. C., McBride, M. L., Spinelli, J. J., Tamaro, S., Gallagher, R. P., & Theriault, G. (2008). Risk of childhood leukemia associated with parental smoking and alcohol consumption prior to conception and during pregnancy: the cross-Canada childhood leukemia study. *Cancer causes & control*, *19*(3), 283–295. https://doi.org/10.1007/s10552-007-9091-8
19. Couto, A. C., Ferreira, J. D., Rosa, A. C., Pombo-de-Oliveira, M. S., Koifman, S., & Brazilian Collaborative Study Group of Infant Acute Leukemia (2013). Pregnancy, maternal exposure to hair dyes and hair straightening cosmetics, and early age leukemia. *Chemico-biological interactions*, *205*(1), 46–52. https://doi.org/10.1016/j.cbi.2013.05.012
20. Couto, A. C., Ferreira, J. D., Koifman, S., Pombo-de-Oliveira, M. S., & Brazilian Collaborative Study Group of Infant Acute Leukemia (2013). Familial history of cancer and leukemia in children younger than 2 years of age in Brazil. *European journal of cancer prevention*, *22*(2), 151–157. https://doi.org/10.1097/CEJ.0b013e3283581d1f
21. Pombo-de-Oliveira, M. S., Koifman, S., & Brazilian Collaborative Study Group of Infant Acute Leukemia (2006). Infant acute leukemia and maternal exposures during pregnancy. *Cancer epidemiology, biomarkers & prevention*, *15*(12), 2336–2341. https://doi.org/10.1158/1055-9965.EPI-06-0031
22. Ross, J. A., Xie, Y., Davies, S. M., Shu, X. O., Pendergrass, T. W., & Robison, L. L. (2003). Prescription medication use during pregnancy and risk of infant

leukemia (United States). *Cancer causes & control : CCC*, *14*(5), 447–451. https://doi.org/10.1023/a:1024953532355

23. Alexander, F. E., Patheal, S. L., Biondi, A., Brandalise, S., Cabrera, M. E., Chan, L. C., Chen, Z., Cimino, G., Cordoba, J. C., Gu, L. J., Hussein, H., Ishii, E., Kamel, A. M., Labra, S., Magalhães, I. Q., Mizutani, S., Petridou, E., de Oliveira, M. P., Yuen, P., Wiemels, J. L., … Greaves, M. F. (2001). Transplacental chemical exposure and risk of infant leukemia with MLL gene fusion. *Cancer research*, *61*(6), 2542–2546.
